# Unprescribed and unnoticed: Retrospective chart review of adverse events of interactions between antidepressants and over-the-counter drugs

**DOI:** 10.3389/fphar.2022.965432

**Published:** 2022-08-29

**Authors:** Jarosław Woroń, Adrian Andrzej Chrobak, Daniel Ślęzak, Marcin Siwek

**Affiliations:** ^1^ Department of Clinical Pharmacology, Chair of Pharmacology, Faculty of Medicine, Jagiellonian University Medical College, Kraków, Poland; ^2^ Department of Anesthesiology and Intensive Care No. 1, Department of Internal Medicine and Geriatrics, University Hospital in Cracow, Kraków, Poland; ^3^ University Center for Monitoring and Research on Adverse Drug Effects in Krakow, Kraków, Poland; ^4^ Department of Adult Psychiatry, Chair of Psychiatry, Jagiellonian University Medical College, Kraków, Poland; ^5^ Division of Medical Rescue, Faculty of Health Sciences with the Institute of Maritime and Tropical Medicine, Medical University of Gdańsk, Gdańsk, Poland; ^6^ Department of Affective Disorders, Chair of Psychiatry, Jagiellonian University Medical College, Kraków, Poland

**Keywords:** antidepressants, drug-drug interactions, over-the-counter drugs, adverse effects, depression

## Abstract

**Aim:** To systematically evaluate prevalence and clinical characteristics of adverse effects of antidepressants and OTC drugs interactions in a retrospective chart review.

**Methodology:** Dataset of 1,145 registered adverse events were evaluated. Reports were selected for further analysis if pharmacoepidemiological avaluation indicated the presence of high probability of a causal relationship between antidepressants and OTC interaction and the occurrence of side effect. Following variables were extracted from the records: sex, age, medical comorbidities, antidepressant and other concomitant medications, clinical consequences ant the possible interaction mechanisms.

**Results:** 368 showed causal relationship with the simultaneous use of antidepressant with another drug. 15 adverse events (4%) were related to the use of OTC medicine, particularly omeprazole, diphenhydramine, Japanese ginkgo biloba, ibuprofen, diclofenac and sildenafil. All of the analysed side effects were categorized as the result of pharmacokinetic interactions. Here we report identified OTC drugs with corresponding antidepressants and clinical manifestations of DDI. Omeprazole: agomelatine (nausea, abnormal dreams), fluoxetine (extrapyramidal symptoms, paresthesias), sertraline (vertigo, yawning), escitalopram (oral vesiculation). Diphenhydramine: sertraline (diaphoresis, insomnia, vertigo), paroxetine (pruritus, headache), duloxetine (oropharyngeal pain). Japanese ginkgo biloba: citalopram (bradycardia), trazodone (vertigo, taste pervesion), mianserine (restless legs syndrome). Diclofenac: escitalopram (oral vesiculation), and fluoxetine (restless legs syndrome). Ibuprofen: agomelatine (anxiety and nausea), sertraline and omeprazole (QTc prolongation). Sildenafil: fluoxetine (genital oedema) and sertraline (myocardial infarction).

**Conclusion:** The use of OTC drugs by the patients should be monitored. Pharmacokinetic interactions between nonprescribed medicines and antidepressants may increase concentration and severity of side effects of latter ones.

## Introduction

The use of over-the-counter (OTC) drugs is an ubiquitous phenomena, and the number of patients undertaking non-prescribed medication is increasing. Studies show that the rate of those practices in developing countries may reach up to 90% (Sánchez-Sánchez et al., 2021). Important role of OTC drugs is to promote self-care and simultaneously decreasing the burden of health care systems (Nomura et al., 2016). However, significant problems may arise when the use of those medications is not properly controlled, and physicians are unaware their patients take them. In up to 46% of cases, medics are not informed about the use of OTC drugs (Albert et al., 2014). Patients may not report and underestimate negative consequences associated with those medicines. Reports indicate that patients consider OTC drugs as safe and notice only their positive effects (Ngo et al., 2010; Eickhoff et al., 2012; Wawruch et al., 2013). The use of those medicines is higher in the group of patients with chronic diseases (Kim et al., 2018) and elderly individuals (Sihvo et al., 2000; Junius-Walker et al., 2007; Albert et al., 2014; Sheikh-Taha and Dimassi, 2018). The lack of information on the use of OTC drugs, especially in the abovementioned groups of patients, carries a significant risk of uncontrolled drug-drug interactions (DDI) leading to harmful effects.

The group of patients particularly vulnerable to the occurrence of side events are those receiving psychopharmacological treatment (Woroń and Siwek, 2018; Woroń et al., 2019; Siwek et al., 2020). Psychotropic medication is commonly used. According to National Health Interview Survey, 15.8% adults were under psychopharmacological treatment in the past 12 months (Terlizzi and Zablotsky, 2019). A group of drugs that are particularly frequently prescribed are antidepressants. In addition to treating major depressive disorder, these medicines are commonly used in therapy of anxiety disorders, eating disorders, insomnia or chronic pain. In the USA during 2015–2018, 13.2% of adults used antidepressants in the past 30 days (Brody and Gu, 2015). In some countries those drugs are also available without prescription from online shops, and they can be purchased from some conventional pharmacies (Sánchez-Sánchez et al., 2021).

Polytherapy, defined as the use of two or more drugs at the same time, is a common phenomenon in clinical psychiatry. In the USA, up to one third of the patients received at least three psychotropic drugs and over the time this proportion is rising (Mojtabai and Olfson, 2010). Simultaneous use of even two medications poses the risk of adverse interactions, and if seven drugs are used at the same time, the occurrence of such an interaction is certain (Vickers et al., 2006; McIntyre et al., 2016; Schatzberg and Nemeroff, 2017; Woroń and Siwek, 2018). This results in the drugs toxicity effects, increased number of adverse reactions and importantly, significant risk of non-compliance (Kukreja et al., 2013). Polytherapy will naturally lead to polypharmacy (polypragmasia) which is defined as the use of multiple concurrent medications, varying from two to eleven drugs at once according to the different definitions (Masnoon et al., 2017). This phenomenon will lead to inadequate and insufficient use of medications what will be associated with the lack of expected efficacy (Woroń and Siwek, 2018). Clinically important group of DDI consists of cytochromes-450(CYPs)-mediated pharmacokinetic interactions. CYPs comprise a large group of enzymes responsible for catalyzing the oxidative biotransformation of most of the drugs. Medicines differ in their interaction profile with CYP enzymes. Through the inhibition of those protein complexes, some drugs can lead to the significant increase of other medicines concentration and their side effects severity (Wienkers and Heath, 2005; Bibi, 2014; Danek et al., 2020).

Given the popularity of both antidepressants and OTC drugs, DDI between those two groups of medicines should be a common phenomenon. However, this topic has not been extensively researched. The aim of our study is to evaluate the incidence and characteristics of adverse interactions of antidepressants and OTC drugs in a retrospective chart review.

## Materials and methods

A retrospective chart review was performed to evaluate the prevalence and clinical characteristics of DDI of antidepressants and OTC drugs. Analysis was performed by all the authors. The dataset involved reports on the occurrence of adverse reactions being the consequence of adverse interactions between simultaneously used drugs. They were evaluated at the University Center for Monitoring and Research on Adverse Drug Effects, Department of Clinical Pharmacology at Jagiellonian University Medical College in Cracow. It is one of the Regional Centers that, in accordance with the legal acts in Poland, formally monitor and report complications related to the pharmacotherapy. Additionally, this organisation provides specialist consultations for both hospitals and clinics from the Lesser Poland, Silesian, Holy Cross and Subcarpathian regions. The Center collaborates closely with the Department of Affective Disorders of the Jagiellonian University Medical College due to the significant increase of the side events associated with the use of psychotropic drugs. Annually, the Center makes approximately 850–1,100 consultations.

**FIGURE 1:**
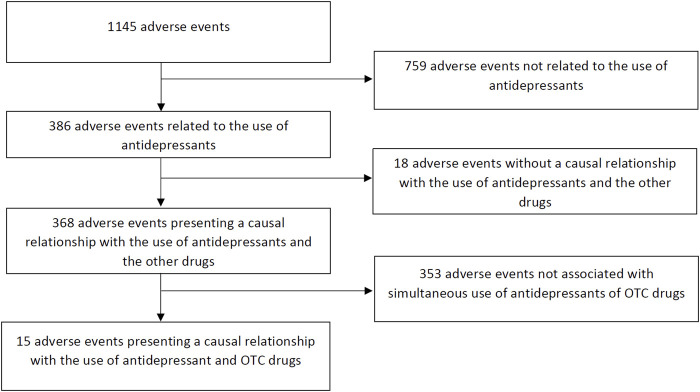
Flow chart of retrospective chart review. OTC—over-the-counter.

In this study we have analysed reports that came from all over the Poland in the period between January 2017 till March 2018. They were selected for further analysis when the following criteria have been met: 1) patient received at least one antidepressant drug, 2) patient used at least one OTC medicine, 3) the presence of a high probability of a causal relationship in terms of pharmacokinetic, pharmacodynamic interactions or the interactions associated with aggregation of side effects associated with the simultaneous use of antidepressant and OTC drugs indicated by pharmacoepidemiological analysis. Figure 1 shows a flow chart of our retrospective chart review. Dataset of 1,145 registered adverse events were analysed. 386 of those were related to the use of antidepressants, from which 368 showed causal relationship with the simultaneous use of antidepressant with another drug. 15 of those adverse events (4.08%) were related to the use of OTC medicine.

## Results

Data extracted from 15 adverse events presenting a causal relationship with the use of antidepressants and OTC drugs is presented in Table 1. The mean age of the patients presented in the reports was 57 ± 10.9. They were seven men and eight women. The most common group of antidepressants associated with the occurrence of adverse events were selective serotonin reuptake inhibitors (SSRIs, 10 cases, 66% cases). Those involved sertraline (seven patients, 47%), fluoxetine (three patients, 20%), paroxetine (one patient, 6%), escitalopram (one patient, 6%), citalopram (one patient, 6%). Other antidepressants related to the occurrence of adverse events were: agomelatine (two patients, 13%), mianserine (one patient, 6%), trazodone (one patient, 6%), duloxetine (one patient, 6%). In case of OTC drugs, interactions involved omeprazole (five patients, 33% - in two cases there was significant interaction of omeprazole with another OTC drug: ibuprofen or diclofenac), diphenhydramine (three patients, 20%), Japanese ginkgo biloba (three cases, 20%), diclofenac (two patients, 13%), sildenafil (two patients, 13%), ibuprofen (two patients, 13%). All of the patients had at least two medical comorbidities. Two patients did not take other medication than antidepressant and OTC drug. All of the analysed side effects were the result of pharmacokinetic interactions. Detailed description of their proposed mechanisms and clinical consequences are presented in Tab.1.

**TABLE 1:** Interactions between antidepressants and over-the-counter drugs in the analyzed group and possible interaction mechanisms.

Antidepressant medication	OTC drug	Sex and age of the patient	Medical comorbidities	Other concomitant medications	Clinical consequences of the interaction	Possible interaction mechanism
Sertraline	Diphenhydramine	F/51	Insomnia, major depressive disorder	-	Diaphoresis, insomnia, vertigo	Pharmacokinetic: inhibition of CYP2D6 by diphenhydramine → increased concentration and side effects of sertraline (metabolized by CYP2D6)
Paroxetine	Diphenhydramine	F/48	Insomnia, major depressive disorder	-	Pruritus, headache	Pharmacokinetic: inhibition of CYP2D6 by diphenhydramine → increased concentration and side effects of paroxetine (metabolized by CYP2D6)
Duloxetine	Diphenhydramine	M/58	Insomnia, major depressive disorder, hypertension	zofenopril, indapamide	Oropharyngeal pain	Pharmacokinetic: inhibition of CYP2D6 by diphenhydramine → increased concentration and side effects of duloxetine (metabolized by CYP2D6)
Citalopram	Japanese ginkgo biloba	M/64	Major depressive disorder, type 2 diabetes, dyslipidemia, vertigo	rosuvastatin, metformin	Bradycardia	Pharmacokinetic: Japanese ginkgo biloba extracts inhibit the activity of CYP3A4 → increased concentration and side effects of citalopram (metabolised by CYP3A4)
Trazodone	Japanese ginkgo biloba	F/62	Insomnia, tinnitus, vertigo, type 2 diabetes	petformin, dapagliflozin	*Vertigo*, taste perversion	Pharmacokinetic: Japanese ginkgo biloba extracts inhibit the activity of CYP3A4 → increased concentration and side effects of trazodone (metabolised by CYP3A4). This is of clinical significance if trazodone is used in daily doses above 300 mg
Mianserine	Japanese ginkgo biloba	F/70	Insomnia, vertigo, hypertension	perindopril, indapamide	Restless legs syndrome	Pharmacokinetic: Japanese ginkgo biloba extracts inhibit the activity of CYP3A4 → increased concentration and side effects of mianserine (metabolised by CYP3A4)
Agomelatine	Omeprazole	M/32	Major depressive disorder, heartburn	antacid on demand	Nausea, abnormal dreams	Pharmacokinetic:omeprazole inhibits CYP2C9 and CYP2C19 → increased concentration and side effects of agomelatine (metabolised by CYP2C9 and CYP2C19)
Fluoxetine	Omeprazole	M/37	Major depressive disorder, gastroesophageal reflux disease	ranitidine	Extrapyramidal symptoms, paresthesias	Pharmacokinetic:omeprazole inhibits CYP2C9 and CYP2C19 → increased concentration and side effects of fluoxetine (metabolised by CYP2C9 and CYP2C19)
Sertraline	Omeprazole	F/64	Major depressive disorder, osteoarthritis of the knee	chondroitin sulfate, diclofenac topical	*Vertigo*, yawning	Pharmacokinetic: omeprazole inhibits CYP2C9 and CYP2C19 → increased concentration and side effects of K/67sertraline (metabolised by CYP2C9 and CYP2C19)
Escitalopram	Diclofenac + Omeprazole	F/67	Major depressive disorder, lower back pain syndrome	pregabaline, etofenamate topical	Oral vesiculation	Pharmacokinetic: omeprazole inhibits CYP2C9 and CYP2C19 → increased concentration of diclofenac, which inhibits CYP2C9 and CYP3A4 → increased concentration and side effects of escitalopram (metabolized by CYP3A4)
Fluoxetine	Diclofenac	F/65	Major depressive disorder, osteoarthritis	paracetamol	Restless legs syndrom	Pharmacokinetic: diclofenac inhibits CYP2C9 and CYP3A4 → increased concentration and side effects of fluoxetine (metabolized by CYP2C9 and CYP3A4)
Agomelatine	Ibuprofen	M/54	Insomnia, lower back pain	pregabaline, buprenorphine	Anxiety, nausea	Pharmacokinetic: ibuprofen inhibits CYP2C9 → increased concentration and side effects of agomelatine (metabolized by CYP2C9)
Sertraline	Ibuprofen + omeprazole	F/59	Major depressive disorder, painful shoulder syndrome, gastroesophageal reflux disease	itopride	QTc prolongation (560 msec)	Pharmacokinetic: omeprazole inhibits CYP2C9 → increased concentration of ibuprofen, which inhibits CYP2C9 → increased concentration and side effects of sertraline (metabolized by CYP2C9)
Fluoxetine	Sildenafil	M/59	Major depressive disorder, dyslipidemia, type 2 diabetes, erectile dysfunctions	atorvastatine, Metformin, pentoxifylline	Genital oedema	Pharmacokinetic: fluoxetine inhibits CYP3A4 → increased concentration and side effects of sildenafil (metabolized by CYP3A4)
Sertraline	Sildenafil	M/64	Major depressive disorder, hypertension, erectile dysfunctions	ramipril lercanidipine	Myocardial infarction	Pharmacokinetic: sertraline inhibits CYP3A4 → increased concentration and side effects of sildenafil (metabolized by CYP3A4)

## Discussion

In this study we have performed the first retrospective chart review of the adverse events caused by the simultaneous use of antidepressants and OTC drugs, based on a thorough analysis of the 1,145 reports. 4% of the adverse events caused by interaction of antidepressants with other drugs, were caused by their simultaneous use with OTC medication, particularly omeprazole, diphenhydramine, Japanese ginkgo biloba, ibuprofen, diclofenac and sildenafil. In this restrospective chart-review, as in our previous studies (Woroń and Siwek, 2018; Woroń et al., 2019; Siwek et al., 2020), the mean age of patients is noteworthy, indicating that the higher risk of DDI is probably age-related.

Omeprazole was the most commonly used OTC drug associated with adverse events evaluated in our study. Proposed mechanisms of those interactions involve interaction with cytochrome 450 isoenzymes responsible for antidepressants metabolism. Particularly, through inhibition of CYP2C9 and CYPC19 omeprazole increases the concentration and side effects of agomelatine (nausea and abnormal dreams), sertraline (vertigo and yawning), fluoxetine (extrapyramidal symptoms and paresthesias) (Karam et al., 1996; Schatzberg and Nemeroff, 2017). In two cases omeprazole was used in conjunction with another OTC drug, which likely contributed to the manifestation of the adverse effects. The first of those patients received escitalopram with omeprazole and diclofenac what was associated with the occurrence of oral vesiculation. This adverse event may be related to the fact that omeprazole inhibits CYP2C9 and CYP2C19 what results in increased concentration of diclofenac. The latter one inhibits CYP3A4 what would lead to increased concentration and side effects of escitalopram, metabolised by this isoenzyme (Schatzberg and Nemeroff, 2017). It has been shown that omeprazole can lead to 93.9% increase of escitalopram concentration, and esomeprazole causes 38,5% increase of sertraline concentration (Gjestad et al., 2015). In cases where it is necessary to combine an antidepressant with a proton pump inhibitor, pantoprazole and lansoprazole will be more favorable. It has been shown that both of those drugs were associated with significantly less pronounced increase of escitalopram and sertraline concentrations (Gjestad et al., 2015).

Diphenhydramine is a first-generation antihistamine drug that acts as an inverse agonist on the H1 receptor with a serotonin reuptake inhibitor property (Khan et al., 2018), which was the root molecule from which fluoxetine was synthesized (Wong et al., 1995). It is most commonly used as a treatment for cold, allergic reactions, as well as insomnia (Simons, 1994). Study has shown that this drug may significantly influence antidepressants metabolism. Lessard et al., 2001 showed that through the inhibition of CYP2D6, diphenhydramine may lead to the more than 2-fold increase in plasma concentration of venlafaxine in the group of extensive metabolizers. This effect could be related to a significantly increased risk of adverse effects (Lessard et al., 2001). In our study, for the first time we have shown increased side effects of other antidepressant drugs, that were simultaneously used with diphenhydramine: sertraline (diaphoresis, insomnia and vertigo), paroxetine (pruritus and headache) and duloxetine (oropharyngeal pain). All of those drugs are metabolized by CYP2D6 (Schatzberg and Nemeroff, 2017), thus it is likely that the mechanisms of their interactions with diphenhydramine will be similar to that for venlafaxine.

Flavones and flavonols contained in the raw Japanese ginkgo biloba material may be potentially used by patients as a self-management aimed to improve brain blood supply, mental performance, memory and to decrease severity of depressive symptoms (Woroń and Siwek, 2018). Drug interactions with herbal OTC drugs are an important problem in psychopharmacotherapy (Woroń and Siwek, 2018). Studies point out that ginkgo biloba have a significant antiplatelet activity, which may add to the antiplatelet effects of SSRIs and SNRIs that may lead to the increased risk of haemorrhagic complications (Ryu et al., 2014; Woroń and Siwek, 2018). Ginkgo biloba may also induce CYP2C19 leading to the accelerated metabolism of omeprazole leading to reduction of its efficacy in prevention of upper gastrointestinal bleeding and may increase the risk of bleeding during SSRI or SNRI therapy (Woroń and Siwek, 2018). Few studies present side effects associated with the use of antidepressants and ginkgo biloba, other than an increased risk of haemorrhage. There is a single case report of coma when this drug was combined with trazodone (Feakes et al., 2000). In our previous study we have shown that simultaneous us of this herbal medicine with fluoxetine was associated with dizziness and hypotension (Woroń and Siwek, 2018). In this retrospective chart review we report three cases of patients with adverse events related to the use of ginkgo biloba, combined with the use of following antidepressants: citalopram (bradycardia), trazodone (vertigo and taste perversion), mianserine (restless legs syndrome). All of those drugs are metabolised by CYP3A4 (Schatzberg and Nemeroff, 2017) which is inhibited by ginkgo biloba (Wang et al., 2022). This may result in the increased concentration and side effects of those antidepressants, involving abovementioned symptoms.

Most of the studies evaluating interactions between antidepressant drugs and NSAIDs are focused on SSRI. Main groups of those interactions involve: 1) inhibition of platelet aggregation and function through the different mechanisms; 2) independent effect of SSRIs without direct pharmacokinetic interaction, e.g. increase of gastric acid secretion; 3) pharmacokinetic interaction through the inhibition of the CYP2C9, leading to the increased concentration of antidepressants metabolised by this isoenzyme (Moore et al., 2015). Most of the studies are focused on the interactions related to the increased risk of heamorrhage. In our chart review, side events associated with the use of NSAIDs were examples of the third group - pharmacokinetic interactions. The use of ibuprofen was associated with an increase in the severity of antidepressants side effects in case of two patients. Combination of this drug with sertraline and omeprazole was related to the significant QTc prolongation (560 msec). To our best knowledge, no cases of a similar interaction have been reported so far. Literature shows that the use of sertraline is associated with the low risk for QTc prolongation (Funk and Bostwick, 2013). Also, studies point out that this SSRI is often recommended as a safe and effective antidepressant in the group of patients with cardiovascular diseases (Mohapatra et al., 2005; Funk and Bostwick, 2013; Woroń et al., 2019). Due to the widespread use of NSAID in this population, special attention should be taken because their use may increase cardiotoxic capacity of the SSRIs. Another adverse event was observed in the case of patient under fluoxetine and diclofenac. Interaction between those two drugs most likely contributed to the manifestation of the restless legs syndrome. Mechanism of exacerbation of this disorder is not fully known yet. It has been hypothesized that fluoxetine, through selective enhancement of serotonin transmission leads to the inhibition of dopaminergic transmission that is related to the restless legs syndrome pathophysiology (Bakshi, 1996; Hoque and Chesson, 2010). We suggest that the occurrence of this side effect in our study was related to the inhibition of CYP2C9 and CYP3A4 by diclofenac. As both of those isoenzymes are involved in the metabolism of fluoxetine, this will increase its concentration and side effects. Finally, we have identified interaction between ibuprofen and agomelatine that resulted in anxiety and nausea. To our best knowledge, this interaction was not described in literature so far. Mechanism of this phenomenon most likely is associated with the inhibition of CYP2C9 involved in agomelatine metabolism (Saiz-Rodríguez et al., 2019).

Erectile impotence is a well-documented, common symptom of depression as well as a side effect of SSRI (Damis et al., 1999; Seidman et al., 2001; Farre et al., 2004). Sildenafil, a phosphodiesterase five inhibitor, is a commonly used drug to treat erectile dysfunctions available as OTC drug in Poland and United Kingdom. It has been shown that this drug may help to ameliorate SSRI-induced sexual dysfunctions as well as those related to the disorder (Damis et al., 1999; Seidman et al., 2001; Nurnberg and Hensley, 2003; Farre et al., 2004). A randomized controlled trial has shown that sildenafil, combined with selective and nonselective serotonin reuptake inhibitors, was well tolerated. The most common adverse event was headache and less frequently flushing, dyspepsia, nasal congestion, and transient visual disturbances. No serious adverse events were reported (Seidman et al., 2001)0. In our study we have identified one case of a patient with myocardial infarction associated with the use of sildenafil combined with sertraline. Sildenafil is considered a safe drug that, when used appropriately, does not seem to increase to risk of myocardial infarction or sudden cardiac death (Kontaras et al., 2008). However, there are case reports indicating occurrence of acute myocardial infarction after sildenafil ingestion in a nitrate free patient without known cardiac history (Feenstra et al., 1998; Kekilli et al., 2005; Hayat et al., 2007). It has been hypothesized that the mechanism of this adverse event may be related to the increased levels of cyclic guanosine monophosphate levels, which mediates the relaxation of vascular smooth muscle, resulting in redistribution of arterial blood flow leading to inadequate coronary perfusion (Feenstra et al., 1998; Kekilli et al., 2005; Hayat et al., 2007). In our case, patient had no previous history of coronary artery disease, and his medical comorbidities involved major depressive disorder, hypertension and erectile dysfunction. We hypothesized that the risk of myocardial infarction could have been increased by the inhibition of CYP3A4 by sertraline, that leaded to increased concentration and side effects of sildenafil. Physicians should take into consideration the occurrence of this rare and serious adverse event related to sildenafil and be aware of pharmacokinetic interactions occurring with sertraline. Another case of adverse event identified in our chart review was a patient that revealed genital oedema after simultaneous use of fluoxetine and sildenafil. We have not found any case of a patient presenting similar clinical consequence of DDI. Most probable mechanism of this adverse event is similar to the previously described patient, and involves increased vasodilatation caused by increase of sildenafil levels due to inhibition of CYP3A4 by fluoxetine.

The most common group of antidepressants associated with the occurrence of adverse events in our retrospective chart review were SSRIs. All of the DDI described in our study represented pharmacokinetic mechanisms related to the inhibition of cytochrome P450 and the increase of those drugs concentrations and side effects. However, it should be noted that the use of SRRIs may associated with occurrence o the serious adverse effect in form serotonin syndrome. Many OTC drugs, such as dextromethorphan, purple echinacea, ginseng or ginkgo biloba (Khan et al., 2018; Woroń and Siwek, 2018) may increase the concentration of serotonin and worsen this life-threatening condition. Thus, doctors should be aware that, apart from pharmacokinetic interactions, concominant use of OTC drugs and SSRIs may be associated with the occurrence of serious adverse events associated with pharmacodynamic reactions.

In this restrospective chart-review, as in our previous studies (Woroń and Siwek, 2018; Woroń et al., 2019; Siwek et al., 2020), the mean age of patients (57 ± 10.9) is noteworthy, indicating that the higher risk of DDI is probably age-related. Studies indicate that older adults are major OTC consumers. Moreover, this group is particularly often affected by the problem of polypharmacy which significantly increases the risk of DDI. National Health and Social Life survey showed that 81% of older adults took at least one prescribed medication, 29% used five ore more drugs. Among them 42% of patients used at least one OTC medicine (Qato et al., 2008; Albert et al., 2014). More frequent use of OTC drugs and more common occurrence of the related DDI in this clinical group may be also associated with the lower average healthy literacy (Albert et al., 2014), presence of medical comorbidities (Sheikh-Taha and Dimassi, 2018), decreased hepatic and prehepatic drug metabolizing efficiency, decreased renal excretory ability, higher sensivity of receptors in central nervous system and deterioration of general homeostatic mechanisms (Turnheim, 2004; Hersh et al., 2007). We have shown, that only 4% antidepressant drug interactions were related to OTC medication. This low detection rate is most likely associated with lack of awareness, rather than rare occurrence of those interactions, what has been also pointed out in the case of studies evaluating prevalence of such drug interactions (Hämmerlein et al., 2007; Scherf-Clavel, 2022). Nationwide survey in Germany showed that only 8.6% of drug-related problems were associated with the use of OTC drugs (Hämmerlein et al., 2007). Another study showed that drug-drug interactions were related to only 4.1% of all drug related problems associated with the use of OTC medicines (Eickhoff et al., 2012). One study showed that even one-third of observed drug-drug interactions may be caused by OTC products (Fiss et al., 2010). Significant number of those interactions may have gone unnoticed because of the lack of documentation of OTC use (Olesen et al., 2013). Considering the frequent use of both antidepressants and OTC drugs, it can be assumed that in clinical practise occurrence of the significant interactions between those two groups of medicines is more common. In order to minimize the observed problem of DDI of antidepressants and OTC drugs, primary care physicians and psychiatrists should ask patients about the use of non-prescribed medications. Also pharmacists may play a role of a strong support group for doctors in reducing the risk of described potential side effects by asking patients about the use of OTC drugs and informing them about possible interactions.

## Conclusion and recommendations

- The pharmacokinetic profile of the patients medications should be investigated in order to evaluate whether there is overlap between cytochrome P450 isoenzymes involved in the metabolism of the drugs used, what may affect their concentration.

- OTC drugs can interact with each other, which may cumulatively increase the concentration and side effects of antidepressants.

- Particular attention should be paid in situations where an antidepressant is used in the maximum dose or the dose is titrated rapidly, because pharmacokinetic interaction with OTC drug may lead to exceeding the therapeutic concentration.

- Interactions of antidepressants and OTC drugs may result of life-threatening adverse events, e.g. myocardial infarction described in our study.

- Patients should be asked by doctors (primary care physician or psychiatrist) as well as the pharmacist about the usage of OTC drugs and informed about possible side effects caused by their simultaneous use with antidepressants.

- The use of OTC drugs by the patient should be described in the medical records in order to be able to monitor the adverse events associated with the use of these drugs.

## Data Availability

The raw data supporting the conclusion of this article will be made available by the authors, without undue reservation.
